# Selenium-Containing Exopolysaccharides Isolated from the Culture Medium of *Lentinula edodes*: Structure and Biological Activity

**DOI:** 10.3390/ijms222313039

**Published:** 2021-12-02

**Authors:** Sandra Górska-Jakubowska, Marzenna Klimaszewska, Piotr Podsadni, Beata Kaleta, Radosław Zagożdżon, Sabina Górska, Andrzej Gamian, Tomasz Strączek, Czesław Kapusta, Marcin Cieślak, Julia Kaźmierczak-Barańska, Barbara Nawrot, Jadwiga Turło

**Affiliations:** 1 Department of Drug Technology and Pharmaceutical Biotechnology, Medical University of Warsaw, 1 Banacha Str., 02-097 Warszawa, Poland; sandra.gorska@wum.edu.pl (S.G.-J.); marzenna.klimaszewska@wum.edu.pl (M.K.); ppodsadni@wum.edu.pl (P.P.); 2 Department of Clinical Immunology, Medical University of Warsaw, Nowogrodzka 59, 02-006 Warsaw, Poland; beata.kaleta@wum.edu.pl (B.K.); radoslaw.zagozdzon@wum.edu.pl (R.Z.); 3 Laboratory of Microbiome Immunology, Ludwik Hirszfeld Institute of Immunology and Experimental Therapy, Polish Academy of Sciences, Weigla 12, 53-114 Wroclaw, Poland; sabina.gorska@hirszfeld.pl; 4 Laboratory of Medical Microbiology, Ludwik Hirszfeld Institute of Immunology and Experimental Therapy, Polish Academy of Sciences, Weigla 12, 53-114 Wroclaw, Poland; andrzej.gamian@hirszfeld.pl; 5 Faculty of Mechanical Engineering and Robotics, AGH University of Science and Technology, Mickiewicza Av. 30, 30-059 Krakow, Poland; tsr@agh.edu.pl; 6 Faculty of Physics and Applied Computer Science, AGH University of Science and Technology, Mickiewicza Av. 30, 30-059 Krakow, Poland; kapusta@agh.edu.pl; 7 Centre of Molecular and Macromolecular Studies, Polish Academy of Sciences, Sienkiewicza 112, 90-363 Lodz, Poland; marcin@cbmm.lodz.pl (M.C.); juliakazmierczak@o2.pl (J.K.-B.); bnawrot@cbmm.lodz.pl (B.N.)

**Keywords:** *Lentinula edodes*, selenopolysaccharides, selenoorganic compounds, Se-exopolysaccharides, mannans

## Abstract

In continuation of our research on the influence of selenium incorporation on the biosynthesis, structure, and immunomodulatory and antioxidant activities of polysaccharides of fungal origin, we have isolated from a post-culture medium of *Lentinula edodes* a selenium (Se)-containing exopolysaccharide fraction composed mainly of a highly branched 1-6-α-mannoprotein of molecular weight 4.5 × 10^6^ Da, with 15% protein component. The structure of this fraction resembled mannoproteins isolated from yeast and other mushroom cultures, but it was characterized by a significantly higher molecular weight. X-ray absorption fine structure spectral analysis in the near edge region (XANES) suggested that selenium in the Se-exopolysaccharide structure was present mainly at the IV oxidation state. The simulation analysis in the EXAFS region suggested the presence of two oxygen atoms in the region surrounding the selenium. On the grounds of our previous studies, we hypothesized that selenium-enriched exopolysaccharides would possess higher biological activity than the non-Se-enriched reference fraction. To perform structure–activity studies, we conducted the same tests of biological activity as for previously obtained mycelial Se-polyglucans. The Se-enriched exopolysaccharide fraction significantly enhanced cell viability when incubated with normal (human umbilical vein endothelial cells (HUVEC)) cells (but this effect was absent for malignant human cervical HeLa cells) and this fraction also protected the cells from oxidative stress conditions. The results of tests on the proliferation of human peripheral blood mononuclear cells suggested a selective immunosuppressive activity, like previously tested Se-polyglucans isolated from *L. edodes* mycelium. The Se-exopolysaccharide fraction, in concentrations of 10–100 µg/mL, inhibited human T lymphocyte proliferation induced by mitogens, without significant effects on B lymphocytes. As with previously obtained Se-polyglucans, in the currently tested Se-polymannans, the selenium content increased the biological activity. However, the activity of selenium exopolysaccharides in all tests was significantly lower than that of previously tested mycelial isolates, most likely due to a different mode of selenium binding and its higher degree of oxidation.

## 1. Introduction

Mushroom-derived polysaccharides are compounds of various structure, variable functionality and diverse biological activity [1]. They are active as antioxidants and have antitumor, immunomodulating, antibacterial, antimicrobial, antiviral, anti-obesity, hypolipidemic, antidiabetic, and hepato-protective properties, among other activities [2,3,4,5,6]. Polysaccharides obtained from higher fungi affect different types of immune responses and are therefore collectively referred to as Biological Response Modifiers (BRM) [7]. In effect, several fungal polysaccharides are used as immunological adjuvants or as a non-invasive form of chemotherapy [8].

Numerous reports in the literature describe the analogous anti-tumor and immunomodulatory effects of some selenium compounds [9,10]. Selenium, as an active center of enzymes involved in oxidative transformations, plays a key role in the resistance mechanisms of plant and animal organisms [11]. A similar pharmacological effect of selenium and fungal polysaccharides, despite the difference in the mechanism, suggests the possibility of hyperadditive synergism (potentiation of action) of both components [12,13,14,15,16,17]. Therefore, it has been hypothesized that the introduction of selenium to the polysaccharide structure would increase the immunomodulatory activity. Although the increased activity of polysaccharides resulting from selenium incorporation has been confirmed by studies conducted in many research laboratories, the mechanism of this action is still unclear [18].

*Lentinula edodes* is a well-known edible and medicinal mushroom, from which lentinan, a branched β-D-glucan with immunomodulatory activity, has been isolated. Since the early 1980s lentinan has been used in several Asian countries as an adjuvant in cancer therapy [19,20,21]. However, some results of current clinical trials of lentinan are inconclusive and do not fully confirm its effectiveness [22,23].

In our previous research, we isolated a selenium-containing polysaccharide fraction from selenium-enriched mycelium of *Lentinula edodes*, using the method described for isolation of lentinan. We obtained Se-polysaccharides with so far undescribed structures: with selenium glycosidically bound in a polyglucoside chain or incorporated into the selenopyranose ring. The results concerning both the structure and the activity of the isolated Se-polysaccharides astonished us. Based on the literature data, we expected a selenated analogue of lentinan, that is (1-6),(1-3)-β-D-glucan, with incorporated selenium atoms, showing a immunostimulatory effect stronger than lentinan alone. However, the isolated Se-polysaccharides displayed unexpected structure and activity: selenated branched (1-3),(1-6)-β-D-glucans, linear (1-6)-β-D-glucans and (1-3)-β-D-glucans, with selective immunosuppressive activity [24,25]. The structure and activity of the isolated mycelial selenopolysaccharides probably resulted from the influence of selenium on polysaccharide biogenesis.

Mycelia of higher fungi may secrete so-called exopolysaccharides (EPS) into the culture medium [26], the secretion of which has been identified in the culture medium by *L. edodes* mycelial cultures [27,28,29]. These EPS have diverse structures and represent both homo- and heteroglycans. They are composed mainly of units of glucose, mannose, galactose and xylose [30]. Interestingly, some of these compounds showed biological activities: e.g., antioxidant, immunomodulating, hypoglycemic, and others [31,32,33,34].

The aim of the present study was to investigate whether supplementation of the culture medium with selenium (in the form of sodium selenite, as in our previous research), would lead to incorporation of this element into the structure of exopolysaccharides secreted into the medium by mycelium of *L. edodes*. The designed research included the study of the primary structure of these compounds, the way selenium binds to the molecule, and the effect of selenation on the biological activity of EPS. To conduct the designed research, we used methods like those we used to study the structure and activity of selenopolysaccharides isolated from the *L. edodes* cell wall [24,25]. We intended to compare the structure, biological properties and, above all, the way selenium binds to the polysaccharide molecule in the Se-exopolysaccharides secreted into the medium by *L. edodes* mycelial cultures with the Se-polysaccharides previously isolated from the mycelium of this fungus. Therefore, we assumed that the same experimental conditions would ensure comparability of previously and currently obtained results.

## 2. Results

### 2.1. Isolation of Selenium-Containing Exopolysaccharides (Se-Exopolysaccharides)

In the case of mycelial *L. edodes* cultures enriched with 30 µg/mL of selenium (added to the medium in the form of sodium selenite), the average efficiency of the isolation of Se-exopolysaccharides from the post-culture medium was 8.1 mg/L. For the reference cultures, cultivated in medium not enriched in selenium, the efficiency of isolation of exopolysaccharides was 25% lower, amounting to 6.1 mg/L. Due to the deproteinization of the culture medium carried out before precipitation of the polysaccharide fractions with alcohol, the obtained result applies only to glycans and proteoglycans with a low share of the protein component.

### 2.2. IR Spectral Analysis

**Figure 1:**
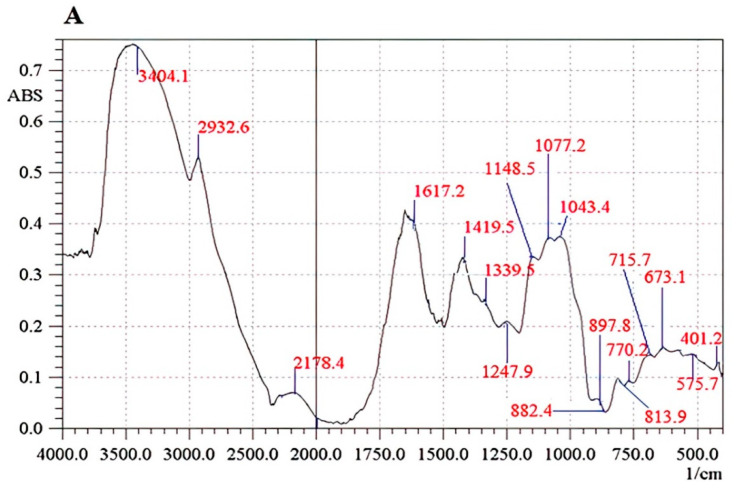

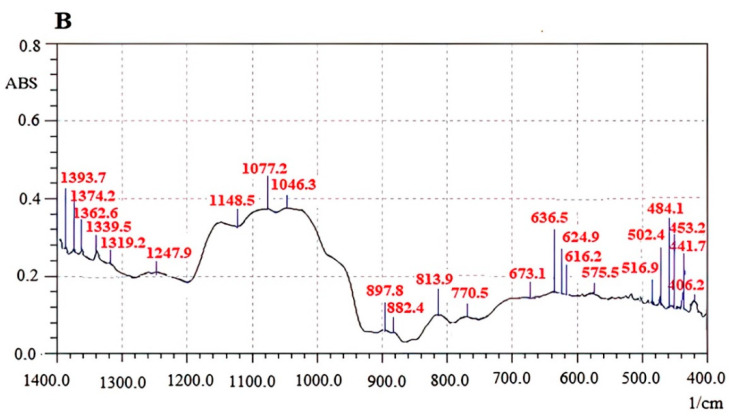
The FT-IR spectrum of the Se-exopolysaccharide fraction Le-P-Se. (**A**) In the scope of 4000.0 to 500.0 1/cm. (**B**) In the scope of 1200.0 to 400.0 1/cm.

**Figure 2:**
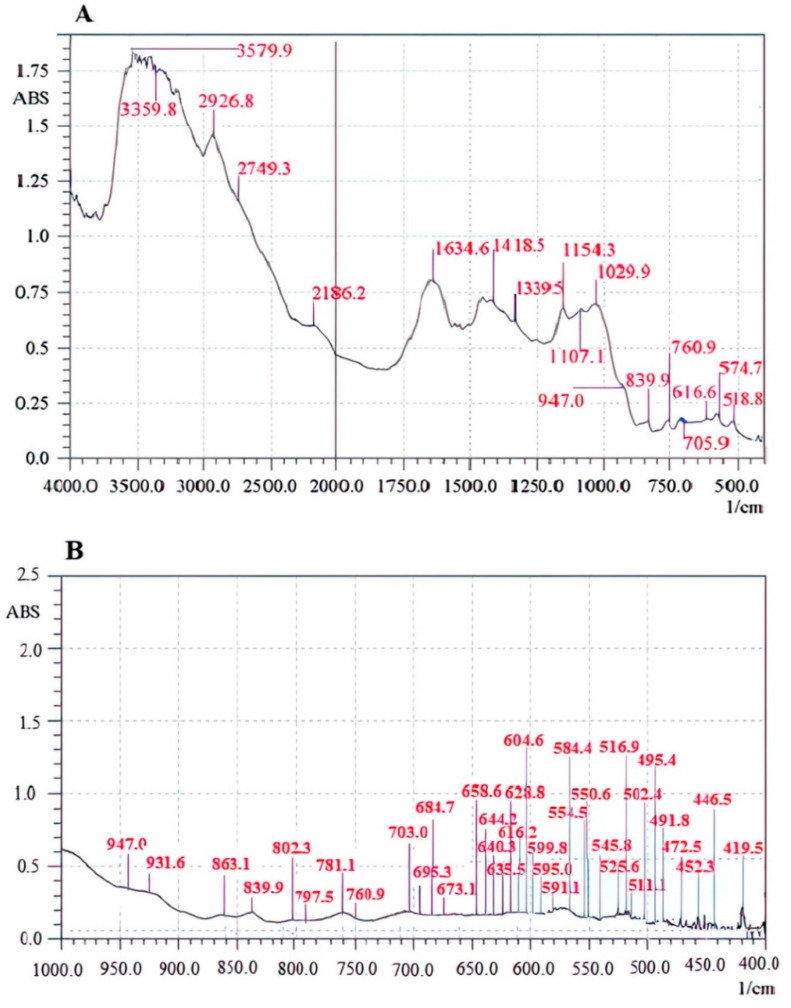
The FT-IR spectrum of the reference exopolysaccharide fraction Le-P-0. (**A**) In the scope of 4000.0 to 500.0 1/cm. (**B**) In the scope of 1000.0 to 400.0 1/cm.

The FT-IR spectrum of the Se-exopolysaccharide fraction (Le-P-Se) is shown in Figure 1, while the FT-IR spectrum of the reference Le-P-0 fraction is shown in Figure 2. 

The IR spectrum of Se-exopolysaccharide Le-P-Se displayed a characteristic band from –OH groups in the range of 3400 cm^−1^. The presence of the –OH stretching signal on the spectrum of reference exopolysaccharide Le-P-0 appeared in the region of 3580–3360 cm^−1^. A signal corresponding to C–H stretching vibrations appeared in the region of 2932.6 cm^−1^ to 2700 cm^−1^. The 1617.2 cm^−1^ peak in the Le-P-Se spectrum and the 1634.6 cm^−1^ peak in the Le-P-0 were assigned to the amide vibrations of proteins [35]. In the 1250–1000 cm^−1^ range, stretching vibrations of C–OH bonds and C–O–C glycosidic bonds dominated. The presence of the bands characteristic of the pyranose ring (1077.2 cm^−1^ and 1107.1 cm^−1^) was found [31,32,33,34,35,36]. The peaks attributed to the vibrations of the γC–O hydroxyl and acetal groups were found around 1339.5, 11,458.5, 1043.4, 715.7 cm^−1^ (Figure 1) and 1339.5, 1154.3, 1029.9, 705.9 cm^−1^ (Figure 2) [37,38,39,40,41]. Based on the analysis of the spectra, both β and α glycosidic bonds were detected. The weaker band at 897.8 cm^−1^ (Figure 1) was specific for β-glucans. However, the band located in the anomeric range at 813.9 cm^−1^ indicated the presence of α-glycosidic bonds: the band bending beyond the CH bond plane in the region of 839.9 cm^−1^ (Figure 2) and 882.4 cm^−1^ (Figure 1), the band of symmetrical skeletal valence vibrations: 770.2 cm^−1^ (Figure 1) and 760.9 cm^−1^ and the band of asymmetric skeletal valence vibrations 947 cm^−1^ (Figure 2) [41,42].

### 2.3. Homogeneity and Molecular Weight of the Exopolysaccharide Fraction

**Figure 3:**
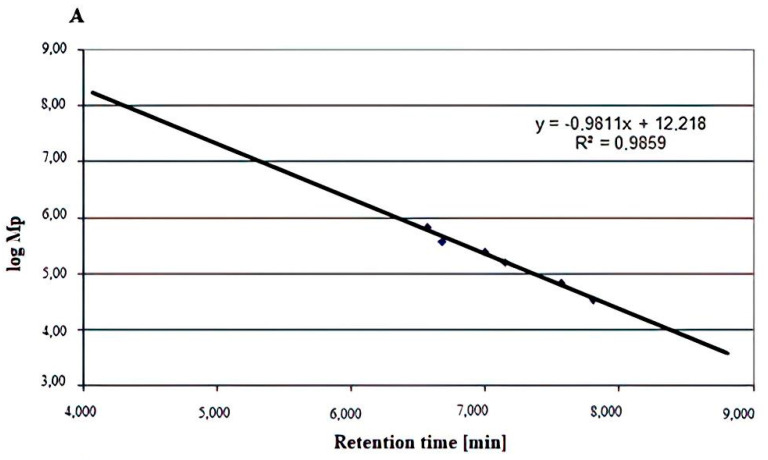

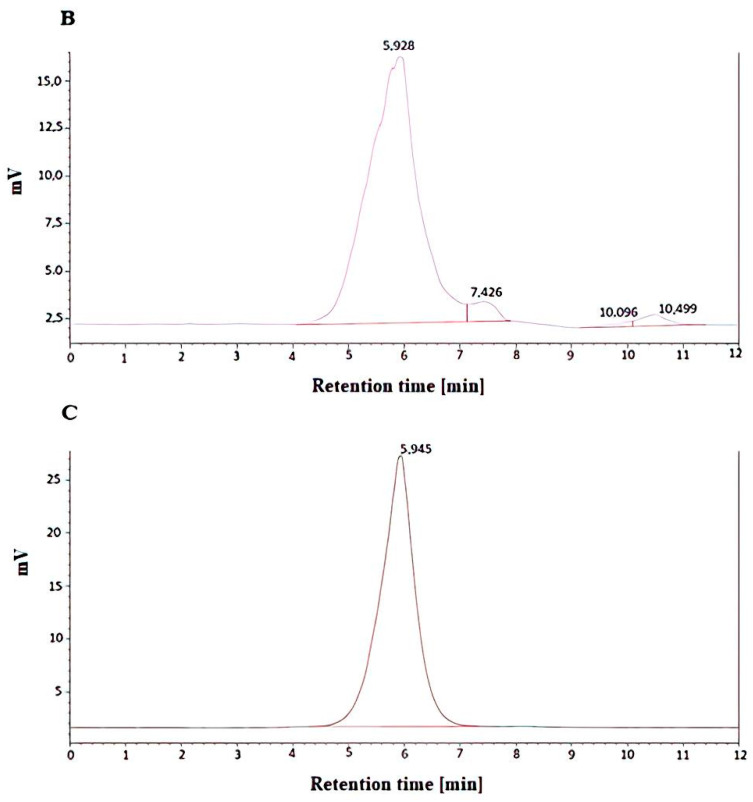
(**A**) Molecular weight distribution of standard β-glucans. (**B**) Chromatogram of the Le-P-0 fraction. (**C**) Chromatogram of the Le-P-Se fraction. Conditions: injection of 20 μL at a concentration of 1 mg/mL of the analyte, using a TSK gel Super Multipore PW-H 6.0 mm ID × 150 mm (TOSOH Bioscience) brand column eluted with ultrapure water (Merck, Darmstadt) as the mobile phase at a flow rate of 0.4 mL/min and conditioned at 50 °C. Mp: molecular weight corresponding to the maximum chromatographic peak.

**Figure 4:**
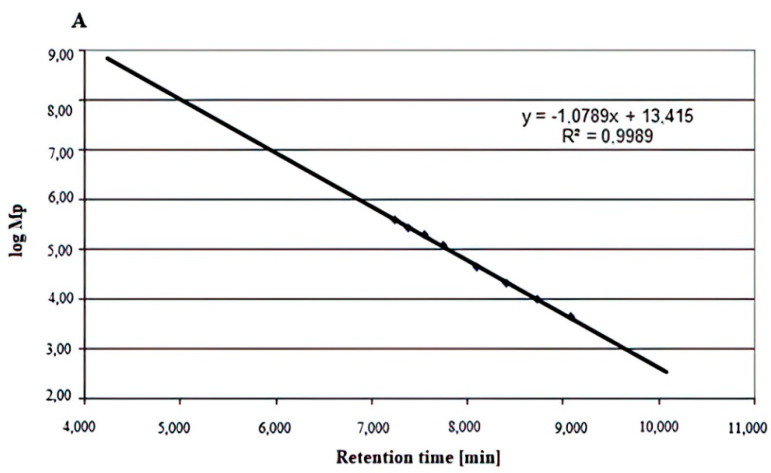

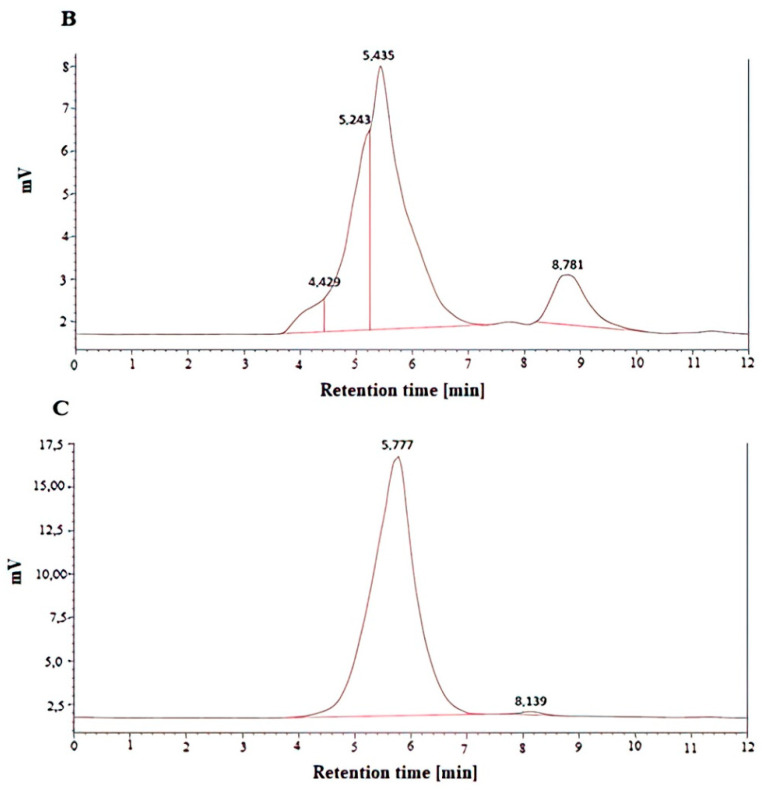
(**A**) Molecular weight distribution of standard α-glucans. (**B**) Chromatogram of Le-P-0 fraction. (**C**) Chromatogram of the Le-P-Se fraction. Conditions: injection of 20 μL at a concentration of 1 mg/mL of the analyte, using a TSK gel Super Multipore PW-H 6.0 mm ID × 150 mm (TOSOH Bioscience) brand column eluted with ultrapure water (Merck, Darmstadt) as the mobile phase at a flow rate of 0.4 mL/min and conditioned at 50 °C. Mp: molecular weight corresponding to the maximum of the chromatographic peak.

The types of glycosidic linkages in the Se-enriched and non-Se-enriched exopolysaccharide fractions were identified by the IR spectra as mainly α- and also β-. Thus, we decided to use both commercially available α- and β-glucan standards (Megazyme) to create the calibration curves obtained by plotting molecular weight (Mp-molecular weight corresponding to that of the maximum of the chromatographic peak) versus time (Figure 3 and Figure 4). The molecular weight of the Le-P-Se fraction determined on grounds of the standard curve for β-glucans (Figure 3) was 2630 kDa, while for the α-glucans, the value was −4468 kDa. Mp values for the reference samples were 954 kDa and 2570 kDa, respectively. The reported result is the mean, the number of replicates (n) was 3, and the relative standard deviation (RSD) was ≤5%.The determined molar mass, however, should be treated as an uncertain value, despite the high value of the correlation coefficients of the standard curves (R^2^ = 0.9859 for β-glucans and R^2^ = 0.9989 for α-glucans), because the elution volume of the examined fractions was significantly lower than that of all commercially available standards. Therefore, the resulting molar mass value was obtained by extrapolation of the calibration curve, resulting in considerable uncertainty of the result. The problem may also arise from the fact that the retention volume in gel permeation chromatography is also influenced (apart from the molar mass) by differences in the spatial structure of the molecules.

### 2.4. The Total Selenium Content

The concentration of selenium in the crude Se-exopolysaccharide fraction Le-P-Se was 582.4 µg/g, whereas in that purified by the Yap and Ng method, it was 219.05 µg/g [43].

### 2.5. Total Protein Content and Amino Acid Composition

**Figure 5:**
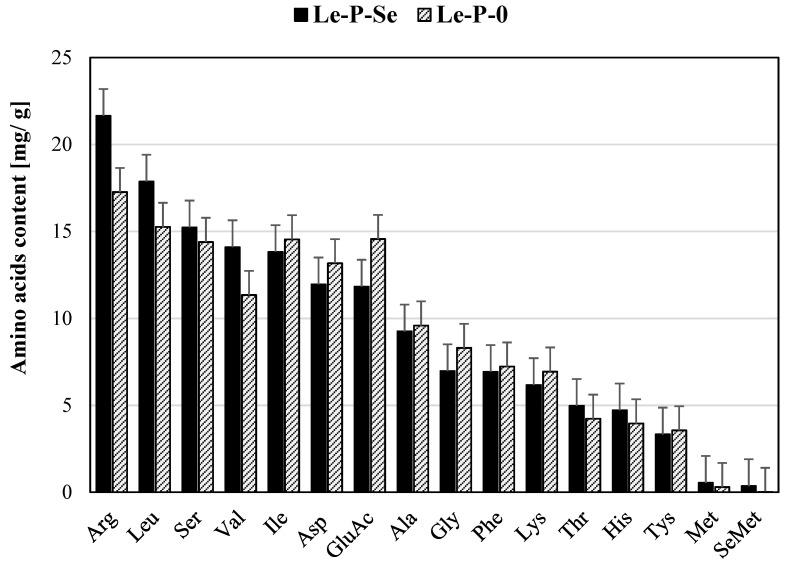
Amino acid composition of Se-exopolysaccharides and the reference fraction.

The total protein content of the Se-enriched exopolysaccharide fraction and the reference (not-Se-enriched exopolysaccharide) was determined by three different methods: the Bradford method, on grounds of the elemental analysis of nitrogen content, and as a sum of the amino acid masses. After the Sevage deproteinization process, the protein content determined by Bradford method in the reference fraction Le-P-Se was lower by 2.23% than in the Le-P-0 fraction and equaled 168.50 mg/g vs. 172.25 mg/g. The percentage of nitrogen in Le-P-Se and Le-P-0 fractions, determined by elemental analysis was 2.88% and 2.98%, respectively, which corresponds to 180.21 mg/g and 186.29 mg/g. The results of determining the content of amino acids in the polysaccharide fractions, after acid hydrolysis, by high-performance liquid chromatography (HPLC), in turn, showed a higher content of amino acids in the selenium-enriched polysaccharide fraction than in the reference (149,671 mg/g vs. 144,625 mg/g). In both exopolysaccharide fractions, the main amino acids in the protein component were: arginine, leucine, serine, and valine (Figure 5). The selenium-containing amino acid selenomethionine was found in both fractions. The content of selenomethionine in the Le-P-Se fraction was significantly higher (0.353 mg/g).

The differences in the results obtained by the different methods of determining the protein content were expected by us, due to the incomplete selectivity of the methods used. Even the chromatographic method of determining the amino acids in the protein hydrolysate is burdened with a certain error, due to the impossibility of determining the proline and hydroxyproline by the OPA method; however, we considered this result to be the most reliable. In summary, it can be assumed that the protein content in the selenium and non-selenated fraction is within 15–18%.

### 2.6. Monosaccharide Composition of Se-Exopolysaccharides

**Figure 6:**
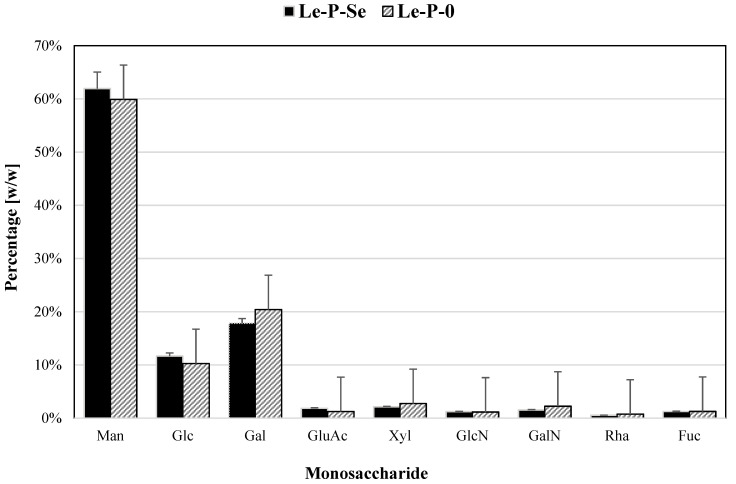
Monosaccharide composition of the crude Se-exopolysaccharide Le-P-Se and the reference fraction Le-P-0.

The monosaccharide composition of the crude Se-enriched exopolysaccharide and the reference fractions was determined after complete hydrolysis with trifluoroacetic acid (TFA) [44]. In both exopolysaccharide fractions, the main monosaccharides were mannose, galactose, and glucose. These three monosaccharides constituted 91.44% of the total amount by weight. In the reference fraction, glucose, mannose, and galactose accounted for 90.57%. The differences in the monosaccharide composition of Se-exopolysaccharides and the reference fraction (Figure 6) were minimal.

After purification of the crude Le-P-Se and Le-P-0 fractions with the Yap and Ng method [43], practically only mannose (89%) with a slight addition of glucose (in the case of the non-selenated fraction) remained in both fractions.

### 2.7. NMR Spectral Analysis

**Figure 7:**
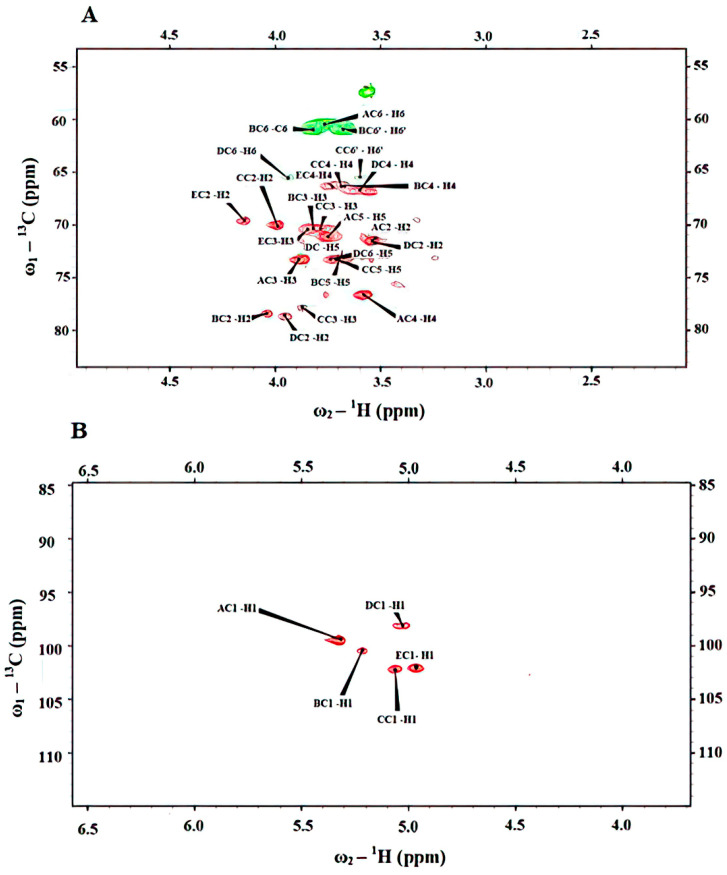
Selected parts of the ^1^H-^13^C HSQC NMR spectra of exopolysaccharides. Selected parts of the ^1^H-^13^C HSQC NMR spectra of exopolysaccharides: (**A**) region of ring carbons (green colors—negative signals for the CH2, red color—positive signals for the CH carbons), (**B**) region of anomeric carbons in the ring.

The chemical shifts were assigned utilizing COSY, TOCSY, NOESY, HSQC, and HMBC experiments (Table 1). Anomeric configurations were assigned on the basis of the chemical shifts of ^3^*J*_H-1,H-2_ and ^1^*J*_C-1,H-1_ values as well as by comparison with previously published NMR data [45,46,47]. Based on the COSY and TOCSY spectrum from the H-2 proton signal for all of the spin systems, it was possible to assign all of the resonances, and from these, all the ^13^C resonances from the HSQC spectrum. The ^1^H-^13^C HSQC-DEPT NMR spectrum (Figure 7) of the Le-P-Se fraction contained five anomeric proton signals: residues A, B, C, D, E at δ_H_ 5.32, 5.22, 5.06, 5.03, 4.97 ppm, respectively, while the same residues correlated with carbon resonances at δ_C_ 99.7, 101.0, 102.8, 99.3, 102.6 ppm, respectively.

Spectra were obtained for ^2^H_2_O solutions at 25 °C, and acetone (δ_H_ 2.225, δ_C_ 31.05 ppm) was used as an internal reference.

Present only in the Le-P-0 exopolysaccharide fraction residue, **A** had the proton chemical signal at δ_H_ 5.32 ppm and the carbon chemical shift at δ_C_ 99.7 ppm and the coupling constants values ^3^*J*_H-1,H-2_ (~3.6 Hz) and ^3^*J*_C-1,H-1_ (~172 Hz). These data suggested that residue **A** was α-linked. The downfield shift of C-4 (76.7 ppm) of **A** indicated that this is a →4)-α-D-Glc*p*-(1→ residue [48,49]. The connectivities were found between C-1 of **A** and C-4 of **A**. We did not observed any cross-peaks between residue **A** and other residues, and thus we determined that this is a separate polymer: α-1,4-glucan. 

Other signals were identified as possessing an α-*manno* configuration (low value of ^3^*J*_H-1,H-2_ and ^1^*J*_C-1, H-1_) and represented the different mannose residues. The downfield shifts of C-2 (79.0 ppm) of residue **B** indicated that it was a →2)-α-D-Man*p*-(1→ residue [50], whereas downfield shifts of C-3 (78.3 ppm) of residue **C** pointed towards it being a →3)-α-D-Man*p*-(1→ residue. Furthemore, residue **D** possessed C-2 (79.1 ppm) and C-6 (66.1 ppm) chemical shifts at low fields owing to glycosylation, and was therefore identified as a →2,6)-α-D-Man*p*-(1→ residue. Likewise, at 4.97 ppm (residue **E**), it was possible to identify non-substituted mannose residues as inferred by their ^1^H and ^13^C resonances. It must be underscored that a very weak signal at the level of the noise appeared at δ_H_/δ_C_ 4.88/99.1 ppm. This signal was proposed to represent another α-mannose, probably →6)-α-D-Man*p*-(1→ according to previously presented mannan data in the literature [51,52]. However, the very low intensity of the signals did not allow identification of the consecutive cross peaks in the NMR spectra, and thus, the interpretation of the linkage types remained ambiguous. The sequence of the monosaccharide residues within the repeating unit of the polysaccharides was obtained by assignment of the inter-residue interactions observed in the 2D NOESY and HMBC spectra. 

**Figure 8:**
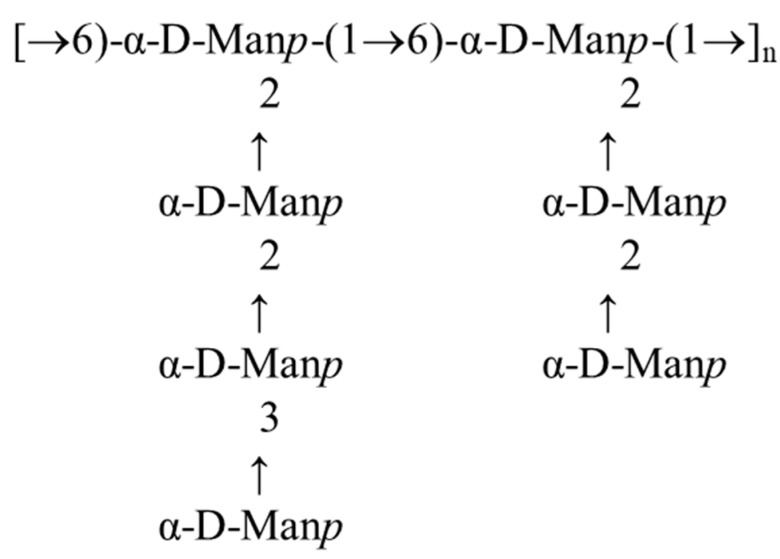
Structure of the highly branched α-D-mannan constituting the main component of the Le-P-Se and Le-P-0 fractions.

The structure was identified as a highly branched α-mannan. The connectivities were found between C-1 of **D** and C-6 of **D**, C-1 of **B** and C-2 of **D**, C-1 of **C** and C-2 of **B**, and C-1 of **E** and C-3 of **C** and C-2 of **B**. Based on the already published mannan polysaccharides [53,54], it was clear that the structure could be presented as follows (Figure 8):

The structures of the selenium-enriched and non-selenium-enriched exopolysaccharide fractions are identical except for the presence of a low amount of α-glucan in the non-selenated fraction. 

### 2.8. X-ray Absorption Spectroscopy (XAS) Determination of Se Oxidation State and Chemical Speciation

**Figure 9:**
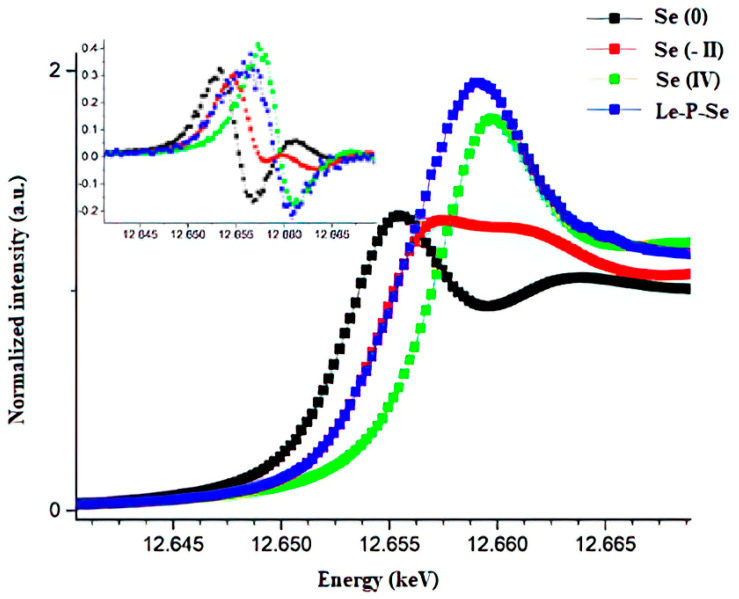
Edge step normalized selenium K edge XANES spectra for the experimental and reference samples. The insert presents their first derivatives: Se (-II)—selenomethionine reference compounds for organically bound Se (-II), Se 0—elemental red selenium powder as reference for Se (0), Se-IV–sodium selenite (Na_2_SeO_3_ as reference for Se (IV)), Le-P-Se—Se-exopolysaccharide fraction.

The exopolysaccharide fraction containing selenium (Le-P-Se) and samples of standard selenium compounds with different degrees of oxidation (reference samples) were analyzed by the XAS method in the X-ray absorption near-edge structure (XANES) range. XANES analysis allows the determination of the average degree of oxidation of an element in the tested sample by determining the energy of the absorption threshold. This energy is related to the chemical shift µ. It is normally defined as the maximum of the first derivative of the dependence of absorption on the energy of the incident beam of radiation (Figure 9). The values of the obtained edge energy shifts in relation to the value measured for the tested sample Le-P-Se, and the reference samples (Se 0, Se II and Se IV) are presented in Table 2.

Comparison of the energy of the absorption threshold of the tested sample against Se 0 and Se IV (E-E0) suggested that selenium in the tested sample is at an average oxidation state of IV and the lower absorption edge energy is related to a different neighborhood than in the sodium selenite. The presence of a strong “white line” (the line at the energy immediately above the absorption edge) suggested that in sample Le-P-Se*,* selenium has oxygen in the immediate vicinity, as in the standard sodium selenite sample. The intensity of the white line is related to the number of empty electron states above the Fermi energy [55]. For the selenomethionine sample, where the closest selenium neighbor is carbon, there is no clear white line, which is related to the degree of covalent bonding that is greater with Se-C than with Se-O.

### 2.9. Extended X-ray Absorption Fine Structure (EXAFS) Analysis of the Local Structure around Se in Se-Exopolysaccharide

**Figure 10:**
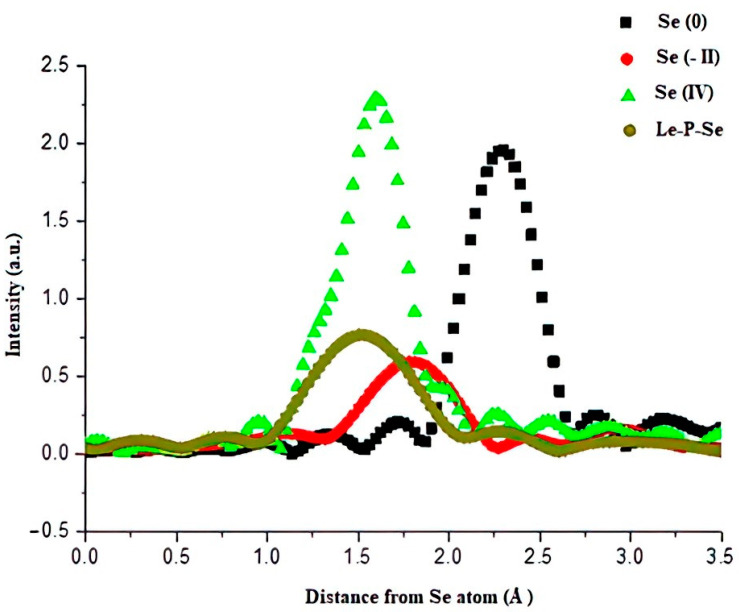
The EXAFS functions χ(R) of the sample studied and the reference materials. Se 0—elemental red selenium powder as reference for Se (0) samples, Se (-II)–selenomethionine reference compounds for organically bound Se (-II), Se-IV–sodium selenite (Na_2_SeO_3_ as reference for Se (IV)), Le-P-Se–Se-exopolysaccharide fraction.

EXAFS analysis provides information about the distribution structure of atoms in the vicinity of the absorbing atom. The analysis uses the phenomenon of oscillation of the absorption coefficient at energies above the absorption threshold due to the presence of adjacent atoms (interference of the emitted photoelectron wave reflected from the atoms with the part originating from the exciting atom). By separating these oscillations from the rest of the signal, one can analyze the “frequencies” of these oscillations that correspond to the distances of neighboring atoms [56]. The EXAFS functions of the tested samples are shown in Figure 10. 

In the tested sample, we observed only one distinct maximum at a distance of 1.5 Å from the absorbing atom. In the selenomethionine reference sample (Se-II), the closest neighbors are two carbon atoms at a distance of about 1.9 Å, while in the sample of sodium selenite (Se IV), the closest three oxygen atoms are at a distance of about 1.3 Å and the next two are at 1.6 Å. The remaining lower maxima are caused by a finite number of measurement points (quantization noise). Taking into account the intensity of the maximum corresponding to the nearest selenium neighborhood in sample Le-P-Se compared to the intensity of the nearest neighbor lines in sodium selenite equals 3 and the fact that the EXAFS signal is stronger in the case of a more rigid network (sodium selenite), it is possible to estimate the number of nearest oxygen neighbors in sample Le-P-Se as 2. 

### 2.10. Biological Activity of Se-Exopolysaccharides and Reference Fractions

#### 2.10.1. The Effects of Exopolysaccharides on HUVEC and HeLa Cells Viability

The results of the MTT test (3-(4,5-dimethylthiazol-2-yl)-2,5-diphenyl tetrazolium bromide test) showed that the cytotoxic activity of selenated (Le-P-Se) and non selenated (Le-P-0) exopolysaccharides isolated from the *L. edodes* culture medium differed markedly. The results are presented in Figure 12. The analysis of the graphs of the viability of HUVEC and HeLa cells incubated with exopolysaccharides showed that the Se-enriched exopolysaccharide Le-P-Se notably prolonged the cell survival time of normal HUVEC cells. The HUVEC cells viability after 24 h was enhanced by 54%, while after 48 h, the value increased to 138%. This effect was not observed for malignant HeLa cells: the viability of the HeLa cells does not significantly change in the presence of both exopolysaccharide fractions, and was even slightly decreased. Interestingly, the reference (not selenated) fraction did not increase the survival rate of HUVEC cells, but even reduced it by 29–47%.

#### 2.10.2. Protective Effect on Exogenous Oxidative Stress

We tested the protective effect of fractions Le-P-Se and Le-P-0 against oxidative stress in cells incubated with hydrogen peroxide. The results showed that Se-enrichment significantly increased the antioxidant effect of exopolysaccharides isolated from the *L. edodes* culture medium (Figure 13). Cell viability in the presence of Se-exopolysaccharide was 12–13% higher as compared to non-selenated exopolysaccharide. When compared to the control (cells treated with hydrogen peroxide at a concentration of 100 µM), the presence of Se-exopolysaccharide increased cell viability by 30%. In turn, when compared to the control cells treated with hydrogen peroxide at a concentration of 300 µM, the increase of cell viability was much higher, reaching 47%. 

#### 2.10.3. Granulocytes Separation and the Effects of Exopolysaccharides on Superoxide Production by Granulocytes

**Figure 11:**
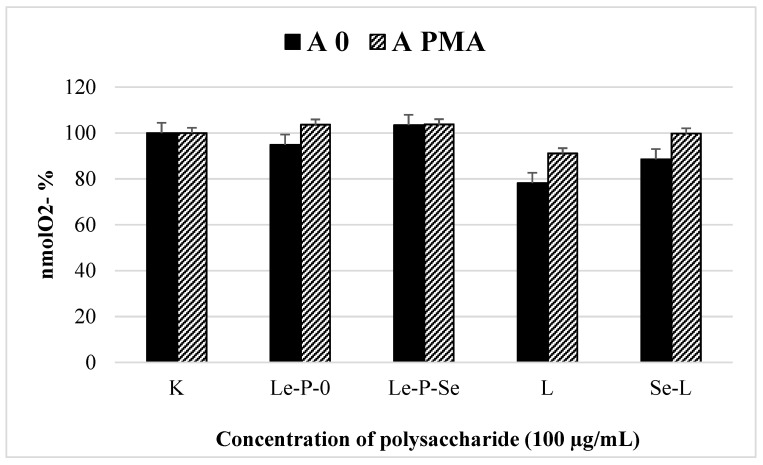
Comparison of the effects of the exopolysaccharide fractions isolated from the culture medium (selenated—Le-P-Se and not selenated Le-P-0) and polysaccharides extracted from the mycelial biomass (selenated—Se-L and not selenated L) [25] on the production of superoxide anions (nmols O_2_^−^). The phorbol 12-myristate 13-acetate (A_PMA_) and the auto- (A_0_) and stimulation of granulocytes was expressed as a level of nmols O_2_^−^, as determined by measurement of cytochrome c reduction rate.

In our previous studies, the influence of all polysaccharides on the production of reactive oxygen species by human granulocytes at concentrations of 1 µg/mL, 10 µg/mL, and even at a high concentration of 100 µg/mL was not observed [25]. Therefore, in the present report, we checked the effect of exopolysaccharide Le-P-Se and Le-P-0 on the production of reactive oxygen species by these cells (Figure 11), but only at a high concentration of 100 µg/mL. The analysis of the concentration of nmol O_2_ in the granulocyte supernatants showed that Le-P-Se and Le-P-0 had no significant effect on the production of reactive oxygen species by these cells (Figure 11). Therefore, it is unfounded to study this effect at lower concentrations.

#### 2.10.4. Comparison of the Effects of Se-Exopolysaccharides on the Proliferation of Human Peripheral Blood Mononuclear Cells (PMBCs)

Both exopolysaccharide fractions Le-P-Se and Le-P-0 tended to reduce PMBC proliferation induced by anti-CD3 mAb (OKT3). The reduction in the number of proliferating T cells, as compared to the control culture, was significant (*p* < 0.05) at the polysaccharide concentrations of 10 and 100 µg/mL. The antiproliferative activity of the Se-enriched fraction was higher than that of the reference fraction, particularly at the concentration of 10 µg/mL (29% vs.14%, Figure 14). At the concentration of 100 µg/mL, the difference in the antiproliferative activity of Le-P-Se and Le-P-0 fractions was lower at 46% versus 41%. 

In all other tests, with non-stimulated PBMCs (autostimulation), stimulation with phytohemagglutinin (PHA) and by use of a suspension of *Staphylococcus aureus* Cowan strain (SAC), no effect on PBMCs proliferation was observed.

## 3. Discussion

The results of numerous studies on the effects of selenium incorporation into polysaccharides have indicated a significant increase in their biological activity, particularly immunomodulating, anti-cancer, and antioxidant effects [18]. In our previous study on the activity of water-soluble Se-polysaccharides isolated from the *L. edodes* mycelium, we also observed significant activity enhancement related to the incorporation of selenium [25]. 

Since exopolysaccharides secreted by mycelial cultures to the culture medium often show strong biological activity and, at the same time, significantly differ in structure from the polysaccharides extractable from the fungal mycelium [33,57], in the current research we undertook structural and activity studies of the Se-exopolysaccharides produced by *L. edodes.* Our first aim was to obtain novel, biologically active seleno-exopolysaccharides with defined structures, while the second aim, no less important, to compare the structural features of Se-exopolysaccharides secreted into the culture medium with previously studied Se-polysaccharides isolated from the mycelium of the same species of fungus [24,25,58]. Numerous researchers have found that the same species of fungus, depending on the strain and growing conditions (e.g., the composition of the medium), may secrete into the medium exopolysaccharides of different structures or in different amounts [59]. Therefore, we tested Se-exopolysaccharides isolated from the post-culture medium of the same *L. edodes* strain, grown under the same conditions and in the same medium as in our previous research. Thus, we were able to reliably compare the structure and activity of Se-exopolysaccharides with Se-polysaccharides isolated from mycelium. We found that supplementing *L. edodes* culture with Na_2_SeO_3_ in relatively high concentration (30 µg Se/mL) [24] resulted in an increase in the production of exopolysaccharides. This outcome was probably caused by the high toxicity of Na_2_SeO_3_, which causes oxidative stress and induces the formation of free radicals. In response to oxidative stress, cells may synthesize non-enzymatic neutralizing metabolites [60]. It can be concluded that EPS may be such a compound [61].

For the purification of the exopolysaccharide fractions, precipitated from the post-culture media, we chose the Yap and Ng method [43]. We have previously used the same method for the purification of the *L. edodes* cell wall Se-polysaccharides [24]. In this method, unlike methods based on the selective precipitation of polysaccharide fractions using strong bases and acids [62], we found no significant losses of selenium: nearly 50%, vs. over 80% when we used the Chihara method (data not yet published). However, in order to overcome the loss of selenium during isolation, the culture was carried out in a medium containing selenium at the relatively high concentration of 30 µg/mL. The selenium content in the crude Se-EPS fraction was 582.4 µg/g, whereas 219.05 µg/g was found in the fraction Le-P-Se purified by the Yap and Ng method [43]. This result indicates the possibility of binding Se to exopolysaccharides in a stable manner, embedding itself in their structure [63]. The selenium content in the Se-exopolysaccharide fraction was slightly higher than in the isolates from the *L. edodes* Se-enriched mycelium (190.8 µg/g) [24].

The successive removal of the protein was performed using the Sevage method [64], which was used before the precipitation of the polysaccharides with alcohol. This process significantly reduced the protein content in the isolated polysaccharide fractions; however, about 15% of the protein component remained in them. The protein component is probably related to the structure of isolated polysaccharides, which are mainly mannans. The formation of connections with proteins is a common phenomenon among fungal polysaccharides [65]. Mannoproteins constitute the outer layer of the fungal cell wall, quite loosely related to its structure; the presence of these compounds in the culture medium is therefore very likely [66,67]. 

There were no significant differences in the monosaccharide composition of Le-P-Se and the reference samples. RP HPLC analysis confirmed that mannose is the basic component of the exopolysaccharides. In turn, in our previous work, we confirmed that glucose was the basic monosaccharide of the extractable mycelial Se-polysaccharides [24]. These results are consistent with the general pattern of fungal cell wall structure. The outer layer, which is gradually released into the environment, is made up of mannans, while the layers located near the plasmolemma contain glucans [68]. In addition to the main components of the exopolysaccharide fraction, i.e., mannose, glucose, and galactose, the monosaccharide composition of *L. edodes* exopolysaccharides is made up of six other types of subunits (Figure 6). This finding suggests that the exopolysaccharide fraction probably also includes complex heteroglycans, which is consistent with literature reports [69]. Uronic acids were found in both fractions, which have a key influence on the antioxidant activity of polysaccharides [70]. Analysis of the polysaccharide structure by IR spectroscopy confirms the results of the RP HPLC analysis of the polysaccharide structure. The IR spectra of the tested fractions (Le-P-Se and Le-P-0) confirmed the presence of mainly α-, but also β-, glycans. Bands characteristic of amido groups indicated the presence of protein components. The structure of the main component of the Se-exopolysaccharide fraction was identified on the grounds of NMR COSY, TOCSY, NOESY, HSQC, and HMBC experiments as a highly branched α-mannan. Its structure is similar to the structure of branched mannans of fungal origin, including yeast, described in the literature [71]. In 2010, Komura et al. hypothesized that the branched α-mannans found in exopolysaccharide fractions of higher fungi originate from the culture medium, often containing yeast extract [66]. However, in the case of mannans isolated by us from submerged cultures of *L. edodes*, this hypothesis does not apply: the yeast mannans described by Komura, present in yeast extracts, have quite low molar masses, compared to our isolates, i.e., about 6.1 × 10^4^ Da. The mannans isolated in our research are characterized by masses of 4 × 10^6^ Da, i.e., many times higher.

We found a significant difference between the currently obtained Se-exopolysaccharides and the Se-polysaccharides previously isolated by us from the mycelium of *L. edodes*: these fractions also contained nearly 16% of α-mannans, but the main components were α and β glucans with dominant 1,4-α-, 1,6-β-, and 1,3-β-glycosidic bonds [24]. 

Another interesting, but still poorly studied, problem is the selenium bonding to the structure of polysaccharides. When the chemical methods are used to enrich natural polysaccharides with selenium, the degree of oxidation of selenium and its bonding ability in Se-polysaccharides are easy to predict [18]. On the other hand, when using biotechnological methods for obtaining the Se-polysaccharides, these important structural features are practically unpredictable and impossible to reliably determine by simple spectral analysis methods (IR, UV, ^13^C and ^1^H NMR, etc.) [57,66]. Thus, one of the important questions that we wanted to answer in the current research was whether the degree of oxidation and the chemical environment of selenium in Se-exopolysaccharides and the mycelial selenopolysaccharides isolated in previous studies are the same. For this purpose, we used, as in previous studies, the XAS (X-ray absorption spectroscopy) method.

To determine the type of interaction between selenium and EPS, the oxidation state of Se was determined and the structure of the distribution of atoms near the selenium atom was verified by X-ray absorption spectroscopy (XAS). The analysis of the spectrum in the X-ray absorption near-edge structure (XANES) showed that in the Le-P-Se sample the absorption energy was close to the Se IV reference sample, but with a difference of 0.8 eV. The average oxidation state in this sample therefore is slightly lower than IV. The exact degree of selenium oxidation cannot be determined due to the different nature of the binding in the Le-P-Se sample than in the Se-II reference sample, as evidenced by the presence of a strong “white line” (Figure 9). The extended X-ray absorption fine structure (EXAFS) simulation analysis showed that the distance to the nearest neighbor is lower than in the case of the mycelium Se-polysaccharides described in our previous publication (fractions Se-L and Se-S) [24]. In those fractions, selenium was clearly bound to two carbon atoms, probably by a glycosidic bond or in the selenopyranose ring [25,30]. In contrast, selenium in the currently examined Se-exopolysaccharide fraction occurs mainly at the fourth oxidation state, but also, similarly to the mycelial polysaccharide fractions, in the second degree. 

The distance for the Le-P-Se sample is similar to the distance of the closest neighbor in the Se IV reference sample where the closest neighbor is oxygen. For the Le-P-Se sample, the spectrum showed only the signal from the closest neighbors, which revealed a large diversity of selenium surroundings (distances of further neighbors) in this sample, causing the signal to fade away from further coordination zones. In summary, in the compounds formed by selenium in this sample, only one closest coordination zone is present, and in it, on average, are located approximately two oxygen atoms. Further atoms are lighter or are at different distances, which causes the mutual extinction of the photoelectron waves reflected from these atoms. 

It is a great challenge to propose a selenopolysaccharide (selenomannose) structure that meets these conditions, so the problem requires further intensive research including molecular modeling. Based on the above data, however, it can be concluded that apart from the mechanism of glycosidically incorporated selenium into the structure of polysaccharides, there are also other interactions between selenites and the structure of exopolysaccharides, resulting in stable selenium derivatives in the IV oxidation state. Based on the available data, we hypothesized that selenium was present in the Se-ester moiety, although it requires confirmation by appropriate studies. Interestingly, selenium in the IV oxidation state was not present in the structure of Se-polysaccharides previously isolated from the mycelial cell wall [24].

A summary of the significant differences in the primary structure, selenium oxidation state and selenium binding between Se-exopolysaccharides secreted into the culture medium and Se-polysaccharides extracted from the mycelium of *L. edodes* [24,25,58] is presented in Table 3.

**Figure 12:**
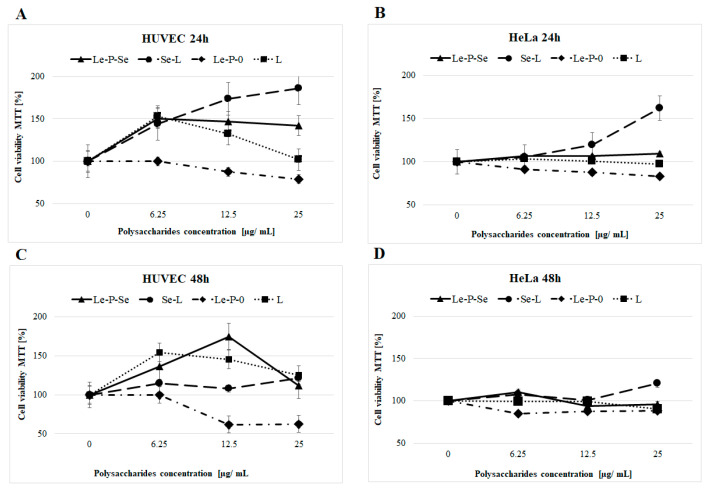
Comparison of the effects of the exopolysaccharide fractions isolated from the culture medium (selenated—Le-P-Se and not selenated Le-P-0) and polysaccharides extracted from the mycelial biomass (selenated—Se-L and not selenated L) [25] on HUVEC and HeLa cells viability. (**A**) HUVEC cells after 24 h of incubation. (**B**) HeLa cells after 24 h of incubation. (**C**) HUVEC cells after 48 h of incubation. (**D**) HeLa cells after 48 h of incubation. The viability of tested cells without added polysaccharide fractions was taken as 100%. The error bars correspond to the standard deviation.

In our previous research, we found that the mycelial *L. edodes* extracts containing Se-polysaccharides showed weak cytotoxic activity in malignant HeLa cells, while strongly increasing HMEC_1_ normal cell viability [72]. Following this, we examined whether these effects were caused by the Se-polysaccharides content [25]. The results showed that both selenated and non-selenated polysaccharides isolated from *L. edodes* mycelium did not adversely affect cell viability [25]. Therefore, we found that selenium compounds other than the Se-polysaccharides were responsible for the previously found cytotoxic activity of *L. edodes* mycelial extracts. The Se-polysaccharide fractions, in turn, were responsible for the previously found opposite effect: a strong increase in cell viability, as determined by the MTT test in human umbilical vein endothelial (HUVECs) and human cervix carcinoma (HeLa) cells. Importantly, this effect was significantly higher (as much as 7-times) for normal cells rather than cancer cells [25]. A similar effect was found for the currently studied Se-exopolysaccharides, but their activity was much weaker. The increase in the survival of normal cells in the MTT test caused by the Se-exopolysaccharides was close to 50% (compared to nearly 90% for selenated mycelial fractions) [25]. The reference fraction showed no such activity, exhibiting even a slight reduction of the viability of the cells. Importantly, Se-exopolysaccharides in contrast to mycelial Se-polysaccharides [25] did not increase the survival of cancer cells (Figure 12A–D).

To summarize, there is a significant difference in the cytotoxic effect in the selected normal and cancer cell lines between the polysaccharide fraction isolated from *L. edodes* mycelium (which is a mixture of α- and β-glucans [24,58] and branched α-mannans isolated from the culture medium. In the first case, there was a tendency to increase the viability of the cells, especially of normal ones, in the second, there was a very weak cytotoxic effect. In both cases, the incorporation of selenium into the polysaccharide molecule significantly increases the viability of normal cells, with less (or no) impact on neoplastic cells. The differences are shown in Figure 12, comparing the activity of the currently obtained Se-exopolysaccharides with the previously obtained Se-polysaccharides isolated from the mycelium of *L. edodes*.

**Figure 13:**
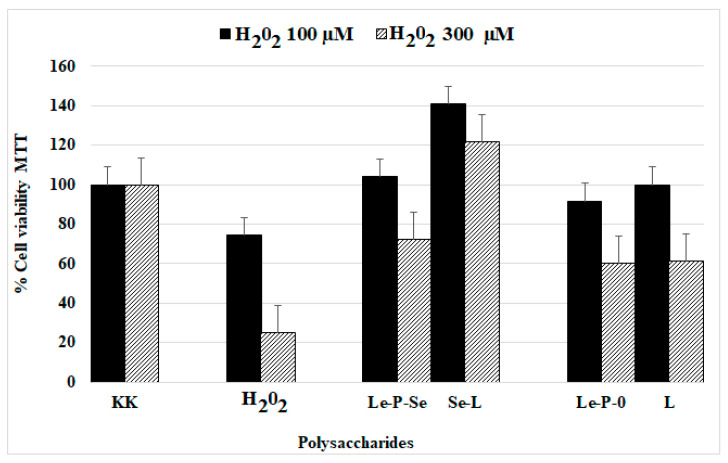
Comparison of the protective effect of exopolysaccharides isolated from the culture medium (selenated fraction Le-P-Se and non-selenated fraction Le-P-0) and polysaccharides extracted from the mycelial biomass (selenated—Se-L and not selenated L) [25] on exogenous oxidative stress induced by hydrogen peroxide in concentrations of 100 and 300 μM. KK—control; H_2_O_2_—control cells H_2_O_2_ treated. The data represent the mean ± S.D of 4 observations.

We found that the Se-exopolysaccharide fraction isolated from the culture medium showed a protective effect on exogenous oxidative stress. Both the Se-exopolysaccharide and the reference fraction displayed antioxidant activity: cell viability in the presence of the selenated exopolysaccharide was higher as compared to the non-selenated fraction and significantly (over two-times) higher as compared to the control, e.g., H_2_O_2_-treated cells (Figure 13). As for the polysaccharide fractions isolated from the *L. edodes* mycelium [25], the effect was more evident for cells exposed to 300 μM H_2_O_2._ However, the Se-exopolysaccharide fraction (Le-P-Se) expressed much lower activity than the Se-polysaccharide fraction isolated from *L. edodes* mycelium (two times vs. six times enhanced cell viability) (Figure 13). The difference in the potency was not evident; however, for the non-selenium enriched fractions: a mixture of α- and β-glucans (L) previously isolated from mycelium and α-mannan (Le-P-0) currently isolated from the culture medium showed similar protective effects against oxidative stress in both tests (Figure 13). There was, however, a significant difference in activity of the selenium-containing polysaccharide fractions isolated from mycelium and from the culture medium (Se-L and Le-P-Se) (Figure 13).

The concentration of selenium in the Se-exopolysaccharide (Le-P-Se) and the previously obtained Se-polysaccharide fraction isolated from mycelium (Se-L) is similar to, or even higher, than that of Le-P-Se (219 vs 191 µg/g). The significant difference in antioxidant activity between selenium-enriched fractions could therefore be related to the differences in the selenium oxidation degree (II in Se-L and IV in Le-P-Se) and a different binding mode of selenium in the structure of polysaccharides.

**Figure 14:**
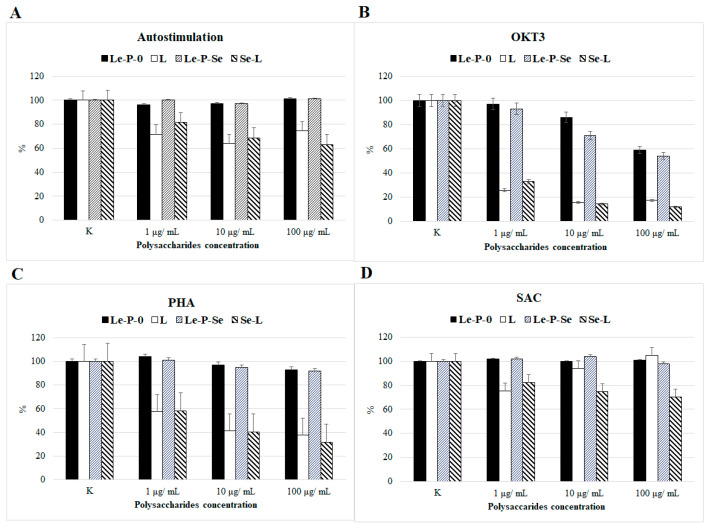
Comparison of the effects of exopolysaccharides isolated from the culture medium (Le-P-Se, Le-P-0) and polysaccharides extracted from the mycelial biomass (selenated—Se-L and not selenated L) [25] on proliferation of peripheral blood mononuclear cells (PBMCs). (**A**) Non-stimulated (autostimulation) and stimulated with: (**B**) Anti-CD3 monoclonal antibody (OKT3); (**C**) Phytohemagglutinin (PHA); and (**D**) Suspension of *Staphylococcus aureus* Cowan strain (SAC). The results are presented as the percentage of proliferation in control cultures (without polysaccharides). The error bars correspond to the standard deviation.

As for the immunomodulatory activity of the tested exopolysaccharide fractions, similarly to the fractions isolated from mycelium, no immunostimulatory activity was found. None of the polysaccharides had a significant effect on the reactive oxygen species generation by granulocytes (Figure 11). However, in tests of the effects of the Se-enriched and reference exopolysaccharide fractions on the proliferation of human PBMCs, similar activity was found as observed for the previously tested mycelial selenopolysaccharides. We observed that both fractions Le-P-Se and Le-P-0 significantly inhibited proliferation of the T lymphocytes induced by anti-CD3 mAb (OKT3), but not by phytohemagglutinin (PHA). Both mitogens are used to evaluate the T proliferative response; however, they use different mechanisms to promote T cell effector functions. As we mentioned in our previous work [25], PHA stimulates T cell proliferation by interactions with the *N*-acetylgalactosamine glycoprotein present on these cells [73]. OKT3 stimulates T cells via CD3-mediated signaling [74]. To summarize, we found that both exopolysaccharide samples, Le-P-Se and Le-P-0, in concentrations of 10 and 100 µg/mL, significantly inhibited proliferation of the T lymphocytes stimulated by OKT3, which suggests their potential effects on the T cell receptor (TCR)/CD3 pathway. This issue warrants further investigations. Notably, the inhibition of T cell proliferation by the exopolysaccharide fractions was distinctly weaker than the effect with the mycelial Se-polysaccharides examined in our previous work (50% vs. 89% of inhibition) [25]. For both exopolysaccharide fractions, Le-P-Se and Le-P-0, no inhibitory effect of polysaccharide fractions on SAC-stimulated PBMC proliferation was observed. This outcome suggested that, similarly to previously tested mycelial fractions Se-L and L [25], currently tested Se-exopolysaccharide fractions may act as selectively immunosuppressive compounds. The effect of Se-exopolysaccharide was significantly stronger than that of the reference fraction, but much weaker than that of Se-polysaccharide isolated from mycelium. The Se-exopolysaccharide was also much more selective: it only affected the proliferation of T lymphocytes and was only active in the test suggesting activity through TCR/CD3. The comparison of the activity of Se-polysaccharides isolated from the culture medium and from mycelium of *L. edodes* (Le-P-Se and Se-L) and non-selenated reference fractions (Le-P-0 and L) is presented in Figure 14.

## 4. Conclusions

### Summarizing the Research Results

We found that the Se-exopolysaccharide fraction secreted into the culture medium by *L. edodes* mycelial cultures consists mainly of highly branched α-mannans of a structure similar to the polysaccharides found in yeast and several higher fungi, but characterized by significantly higher molecular weight. Selenium in the Se-exopolysaccharide fraction was bound to the structure of polysaccharides in a different way than seen in the Se-polysaccharides extracted from *L. edodes* mycelium and showed the oxidation state IV. The isolated Se-mannans show basically similar biological activity to the selenium-enriched fractions previously isolated from the *L. edodes* mycelium, which were mainly α- and β-glucans, but also contained the α-mannan moiety. The effect of Se-exopolysaccharides, in the cytotoxicity (MTT) test, included selective enhancement of the survival of normal cells, with no effect on cancer cells. The Se-polysaccharides isolated from mycelium, in turn, showed much stronger, but non-selective effects in this test. The isolated Se-exopolysaccharide showed high antioxidant activity, significantly higher than that of the reference fraction, but much lower than that for the Se-polysaccharide fraction isolated from mycelium. The Se-exopolysaccharide showed a similar inhibitory effect on the proliferation of T lymphocytes as observed for the previously tested mycelial Se-polysaccharide, but only in the OKT3 test, without the simultaneous effect on B lymphocytes. The effect was much weaker than that for the fraction isolated from mycelium, although more selective. The differences between the activity of the seleno-exopolysaccharides and selenopolysaccharide fractions isolated from the mycelium were probably to a significant extent due to the difference in selenium binding and its degree of oxidation. Both the structure and the mechanism of the potential immunosuppressive effect of the studied Se-exopolysaccharides, composed of selenium-containing highly branched α-mannans with a 15% protein component, require further detailed studies.

Here we summarize the main findings presented in the current paper, and point out the main differences in the structure and biological activity of the Se-exopolysaccharides secreted into the culture medium by *L. edodes* mycelia and Se-polysaccharides isolated from the *L. edodes* mycelial cell wall (described in our previous research) [24,25,58]. We have shown that, unlike linear Se-1,4-α-D-glucans, Se-1,3-β-D-glucans, Se-1,6-β-D-glucans and branched 1,3-β-1,6-β-D-glucans isolated from the mycelial cell wall, the Se-exopolysaccharides secreted into the medium are highly branched Se-1,2-α-1,4-α-mannans.

- In contrast to the cell-wall Se-polysaccharides (Se-glycosides or selenopyranose-derivatives, with selenium in the -II oxidation state), selenium was bound to Se-exopolysaccharides most likely by ester bonds, remaining in the IV oxidation state. Interestingly, this oxidation state was absent in cell wall Se-polysaccharides.
- We have also shown a significant effect of the different polysaccharide structures and degrees of selenium oxidation on the immunomodulating, cytotoxic, and antioxidant properties of Se-polysaccharides. However, this activity was not completely different or the opposite to previously described, while mannans are in general present in the outer layers of the cell wall. They are secreted into the culture medium, but also remain in small amounts in cell wall isolates.

Due to the potential use of the Se-polysaccharides derived from *L. edodes* as novel immunosuppressive drugs in transplantology and autoimmune diseases, we plan to conduct advanced research on the mechanisms of action and pharmacokinetic properties (ADME Tox studies) of these compounds.

## 5. Materials and Methods

### 5.1. Microorganism and Cultivation Media

The *L. edodes* (Berk.) Pegler strain used in this study was American Type Culture Collection (ATCC) 48085. Se-Exopolysaccharide fractions (Se-EPS), hereafter referred to as Le-P-Se, were isolated from the post-culture liquid medium, in which the submerged culture of this fungus was carried out. The mycelial cultures were grown under the conditions described in our previous reports [24,75,76]. To specify, the mycelial cultures were cultivated under submerged conditions in a 10 L fermenter (BioTec FL 110, Stockholm, Sweden.). The culture media were fortified with selenium at a concentration of 30 μg/mL, by the addition of sodium selenite (Na_2_SeO_3_, Sigma, Cell Culture Tested). The initial pH of the medium was 6.5. The medium was inoculated with 5% (*v*/*v*) of seed culture and cultivated at 26 °C. Fermentation was performed for 10 days under the following conditions: aeration rate, 0.5 vvm (volume per volume per minute); agitation speed, 200 rev/min; and working volume, 8 L. Mycelia were filtered off and the liquid post-culture medium was used for the isolation of the Se-exopolysaccharides. At the same time, the reference cultures were grown under the same conditions in media not enriched with selenium. The post-culture media were used for the isolation of reference exopolysaccharides (Le-P-0).

### 5.2. Isolation and Purification of Se-Enriched and Reference Exopolysaccharide Fractions

The Se-exopolysaccharide (Le-P-Se) and reference (Le-P-0) fractions were isolated from the Se-enriched and reference post culture liquid medium. The culture medium was concentrated to 20% with rotary evaporators (Buchi Rotavapor R-200). After cooling the concentrate to room temperature, the solution was deproteinized for 10 min by the Sevage method [64] with a mixture of chloroform and butanol (3:1 *v*/*v*). The deproteinization procedure was repeated 3 times. Then the exopolysaccharides were precipitated by adding one volume of 96% ethanol 1:1 and stored for 14 days at 4 °C. The precipitate was centrifuged to separate the supernatant from the pellet (10 min, 6800 rpm). The pellet was frozen in liquid nitrogen at −195 °C and subjected to lyophilization. The freeze-dried crude exopolysaccharide fractions were purified by use of the modified Yap and Ng method [43] described in our previous paper [24]. Namely, hot water (60 °C, 30 volumes (*v*/*w*)) was added to the freeze-dried pellet and homogenized for 1 min. The homogenate was boiled for 10 h on a magnetic stirrer to obtain a clear solution. The hot solution was passed through a vacuum filter. After cooling the extract to room temperature, ethanol (1:1, one volume) was added and the mixture was stored for 14 days at 4 °C. The centrifugation, freezing in liquid nitrogen, and freeze-drying steps were repeated to obtain purified exopolysaccharide in powder form.

### 5.3. Structural Analysis of Exopolysaccharide Fractions

#### 5.3.1. IR Spectral Analysis

IR spectra were recorded in the range 4000–700 cm^−1^ with a Fourier transform infrared (FTIR) spectrometer (Shimadzu, FTIR 8300). The test samples were prepared using the KBr-disk method [77].

#### 5.3.2. Determination of the Molecular Weight by High Performance Gel Permeation Liquid Chromatography (HPGPC) with Light-Scattering Detection

The molecular weight and homogeneity of the polysaccharide fractions were determined by size exclusion chromatography (HPSEC) with light scattering (ELSD) detection according to the procedure reported by Cheong et al. (2015) [77], with modifications. The GPC system (Shimadzu) is equipped with two HPLC pumps (Shimadzu), the GPC TSKgel SuperMultipore PW-H column, 6.0 ID × 150 mm, (TOSOH Bioscience), Guard SuperMP PW-H 4.6 mm ID × 35 mm (TOSOH Bioscience), column oven (Shimadzu) and evaporator lamp–scattering detector (ELSD LTII). Deionized water was used as the mobile phase. Column and detector temperatures were 40 °C and 50 °C, respectively, and the flow rate and injection volume were 0.4 mL/min and 20 µL, respectively. The β-glucan standards (40 kDa, 123 kDa, 183 kDa, 245 kDa, 359 kDa, Megazyme, Ireland and 500 kDa, Boc Sciences, New York USA) and α-glucan standards (401 kDa, 277 kDa, 196 kDa, 124 kDa, 43.5 kDa, 21.4 kDa, 9.9 kDa, 4.4 kDa, PSS Polymer Standards Service Gmbh) were used to plot a calibration curve. A calibration curve was plotted as the correlation between the retention time and the molecular weight (Mp) of the standards.

#### 5.3.3. Reversed Phase High Performance Liquid Chromatography (RP-HPLC) Determination of Se Content

The RP-HPLC procedure used was a modified fluorometric method of Se determination after derivatization with 2,3-diaminonaphthalene (4,5-benzopiazselenol formation) with fluorescence detection [78], as described in our previous papers [63,75,76]. 

#### 5.3.4. Determination of Protein Content

The results were obtained by the Bradford spectrophotometric method [79] and the nitrogen content and fractions analysis with the CHNS Vario EL III elemental analyzer (Elementar, Germany). Protein content was calculated using a conversion factor of 6.25 from total nitrogen to protein.

#### 5.3.5. Reversed Phase High Performance Liquid Chromatography (RP-HPLC) Determination of the Amino Acid Composition of a Protein Component after Acid Hydrolysis

The derivatization of amino acids with the OPA reagent was performed. The amino acids in the dry matter of the polysaccharide hydrolysate were then determined by high-performance liquid chromatography. Hydrolysis was performed prior to analysis under acidic conditions with 6 M HCl at 110 °C for 24 h. The RP HPLC gradient method and the derivatization reaction conditions have been described in the previous work [75].

#### 5.3.6. Reversed Phase High Performance Liquid Chromatography (RP-HPLC) Determination of Monosaccharide Composition

Monosaccharide composition of the polysaccharides was determined by a reversed phase high performance liquid chromatography (RP HPLC) method described in our previous paper [44]. Prior to the analysis, the exopolysaccharides were hydrolyzed for 5 h with 3 M TFA at 120 °C.

#### 5.3.7. X-ray Absorption Spectroscopy (XAS)

XAS measurements were carried out at beamline X of the synchrotron laboratory HASYLAB DESY in Hamburg, Germany. The selenium K edge spectra were collected in fluorescence mode at the temperature of 10 K to reduce the influence of thermal disorder on the extended X-ray absorption fine structure (EXAFS) spectra. The following reference compounds were used: selenomethionine for organically bound Se (-II) (Aldrich), red selenium powder obtained by the reduction of selenites at the Department of Drug Technology and Pharmaceutical Biotechnology, Medical University of Warsaw for Se (0), sodium selenite (Na_2_SeO_3_, Sigma, Cell Culture Tested, Sigma, Saint Louis, MO, United States)for Se (IV). For the X-ray absorption near-edge structure (XANES) analysis, the XAS experimental data were normalized to the edge step and the backgrounds were subtracted using the standard procedures in the ATHENA program. EXAFS simulations were carried out using FEFF 8.4 code.

#### 5.3.8. NMR Spectral Analysis

The NMR spectra were obtained on a Bruker 600 MHz Avance III spectrometer using a 5 mm QCI ^1^H/^13^C/^15^N/^31^P probe equipped with a z-gradient. About 10 mg of exopolysaccharides were dissolved in ^2^H_2_O (99.96%, 0.6 mL). The NMR spectra were recorded at 25 °C using acetone (δ_H_ 2.225, δ_C_ 31.05 ppm) as an internal reference. The data were acquired and processed using Bruker Topspin software (version 3.1) and SPARKY (Goddard and Kneller, 2001). The signals were assigned using one and two homo- and heteronuclear experiments: COSY, TOCSY, NOESY, HSQC with and without carbon decoupling and HMBC. The TOCSY experiments were carried out with mixing times of 30, 60 and 90 ms, NOESY with mixing times of 100 ms and 300 ms, and HMBC with 60 ms mixing time.

### 5.4. Biological Activity of Se-Exopolysaccharides and Reference Fractions

#### 5.4.1. The Effect of Exopolysaccharides on HUVEC and HeLa Cells Viability (MTT Assay)

The methodology was consistent with that previously described by Kaleta et al. [25]. Cell viability was determined by the MTT assay. The activities of the exopolysaccharides isolated from selenium-enriched medium (Le-P-Se) and from non-selenium medium (Le-P-0) were tested.

#### 5.4.2. Protective Effect on Exogenous Oxidative Stress

The methodology was consistent with that previously described by Kaleta et al. [25]. The HeLa cells were incubated with Le-P-Se and Se-P-0 exopolysaccharide fractions at a concentration of 25 μg/mL for 30 min. H_2_O_2_ at a concentration of 100 µM or 300 µM was then added to the cells for 24 h.

#### 5.4.3. Granulocyte Separation and the Effects of Exopolysaccharides on Superoxide Production by Granulocytes

Blood samples (8 mL) were collected from healthy blood donors. All blood samples were commercially obtained from the Regional Blood Centre in Warsaw. Isolation of granulocytes was performed by density gradient centrifugation on Histopaque-1077 and Histopaque-1119 (Sigma). Granulocytes were harvested from the interface between Histopaque-1077 and Histopaque-1119. Granulocytes (2.5 × 10^5^ cells/well) were cultured in 96-well round-bottom microplates in the medium containing PBS, 6 mM glucose (Sigma) and 1% bovine serum albumin (BSA, Biowest). Granulocytes were either activated or not by phorbol 12-myristate 13-acetate (PMA, Sigma) and incubated with cytochrome c (Sigma) and exopolysaccharides fractions at the concentration of 100 μg/mL for 30 min at 37 °C and 5% CO_2_ in a humidified incubator. Control cultures contained an equivalent amount of medium. Generation of O_2_^–^ by reduction of cytochrome c was detected at room temperature using a microplate reader at 550 nm (Chromate 4300 Microplate Reader). Because cytochrome c may be reduced by other radical species, additional control experiments with superoxide dismutase (SOD, 30 mg/mL, Sigma) were performed to confirm that the reduction was dependent on O_2_^–^. All experiments were performed in triplicates.

The study was approved by the Local Ethics Committee (no. KB/174/2017) and all subjects provided written informed consent. The procedures followed were in accordance with the Helsinki Declaration of 1975, as revised in 2000.

#### 5.4.4. PBMC Separation and the Effects of Polysaccharides on Mitogen-Stimulated PBMC Proliferation

Blood samples (8 mL) were collected from healthy blood donors. Isolation of peripheral blood mononuclear cells (PBMCs) was performed by density gradient centrifugation on Histopaque-1077 (Sigma). All blood samples were commercially obtained from the Regional Blood Centre in Warsaw. The separated PBMCs were resuspended in RPMI 1640 medium (Gibco) containing 2 mM L-glutamine (Sigma), antibiotic-antimycotic solution (1.5% penicillin-streptomycin-amphotericin, Invitrogen) and 10% fetal bovine serum (FBS, Gibco). PBMCs (1 × 10^6^ cells/well) were cultured in 96-well flat-bottom microplates. Cells were either not stimulated or stimulated with mitogens: anti-CD3 mAb (OKT3, 1 μg/mL, BD Pharmingen), phytohemagglutinin (PHA, 20 μg/mL, Sigma) and a suspension of *Staphylococcus aureus* Cowan strain (SAC, 0.004% *w*/*v*, Calbiochem) and incubated with Le-P-Se or Le-P-0 exopolysaccharide fractions at concentrations of 1, 10 and 100 µg/mL. PBMCs were cultured for 72 h at 37 °C and 5% CO_2_ in a humidified incubator. PBMC proliferation was assessed using [^3^H]-thymidine (1 μCi/well; 113 Ci/nmol, NEN) incorporation. After 18 h of culture, PBMCs were harvested and the radioactivity was measured with the scintillation beta-counter (Wallac, PerkinElmer). The amount of radioactivity incorporated into DNA of PBMCs was proportional to the number of proliferating cells. The readout was expressed as corrected count per minute (ccpm) per well. All experiments were performed in triplicates.

### 5.5. Statistical Analysis

The Mann–Whitney U-test and Spearman correlation were applied using Statistica 9.0 (StatSoft Inc, Tulusa, Ok, United States)Differences from control cultures were considered statistically significant at a *p* value < 0.05.

## Figures and Tables

**Table 1:** ^1^H and ^13^C NMR chemical shifts of sample Se-exopolysaccharides fraction.

	Chemical Shifts ^1^H, ^13^C (ppm).						
	H1	H2	H3	H4	H5	H6	H6′
Sugar residue	C1	C2	C3	C4	C5	C6	
A	5.32	3.54	3.88	3.58	3.75	3.75	
→4)-α-D-Glc*p*-(1→	99.7	71.5	73.3	76.7	71.1	60.4	
B	5.22	4.04	3.82	3.67	3.70	3.82/3.66	5.22
→2)-α-D-Man*p*-(1→	101.0	79.0	70.7	66.7	73.8	61.5	
C	5.06	3.98	3.86	3.68	3.71	3.82/3.66	
→3)-α-D-Man*p*-(1→	102.8	70.6	78.3	66.7	73.9	61.5	
D	5.03	3.95	3.78	3.60	3.94	3.94/3.59	
→2,6)-α-D-Man*p*-(1→	99.3	79.1	70.8	67.2	73.7	66.1	
E	4.97	4.14	3.85	3.73	3.68	3.82/3.66	
α-D-Man*p*-(1→	102.6	70.1	70.9	66.8	73.4	61.5	

**Table 2:** Edge energy values for the experimental samples and reference samples.

Sample	E	E-E0
Le-P-Se	12,656.9	3.6
Se 0	12,653.3	0.0
Se-II	12,654.6	1.3
Se IV	12,657.5	4.2

E—Edge energy values for the experimental samples, E0—Edge energy value for the elemental red selenium sample, Le-P-Se–Se-exopolysaccharide fraction, Se(-II)–selenomethionine reference compounds for organically bound Se(-II), Se 0—elemental red selenium powder as reference for Se(0), Se-IV–sodium selenite (Na_2_SeO_3_ as reference for Se (IV)).

**Table 3:** Structural differences between the Se-exopolysaccharides secreted into the culture medium and Se-polysaccharides extracted with hot water from the mycelium of *L. edodes*.

Characteristic Feature	Cell Wall Se-Polysaccharides [24,25,58]	Culture Medium Se-Exopolysaccharides
Protein content	8–10%	15–18%
Monosaccharide composition (main components)	82% Glucose 13% Mannose	89% Mannose 5% Glucose
First order structure of main components	Linear 1,4-α-glucans, linear 1,3-β- and 1,6-β-glucans and 1,3-β-branched 1,6-β-glucans	Highly 1,2-α-branched 1,4-α-mannans
Selenium content	190 µg/g	219 µg/g
Type of selenium binding	Se-glycosidic bound, most likely in 1,4-α- or 1,3-β-Se-glycosides, or in the Se-pyranose ring	Most likely in Se-ester bound: -Se=O(OH)
Main Se-oxidation state	II	IV
Molecular weight (approx.)	3630 kDa	4468 kDa

## Data Availability

The data presented in this study are available on request from the corresponding author and co-authors.
